# Effects of bacterial cultures, enzymes, and yeast-based feed additive combinations on ruminal fermentation in a dual-flow continuous culture system

**DOI:** 10.1093/tas/txab026

**Published:** 2021-02-11

**Authors:** S L Bennett, J A Arce-Cordero, V L N Brandao, J R Vinyard, B C Agustinho, H F Monteiro, R R Lobo, L Tomaz, A P Faciola

**Affiliations:** 1 Department of Animal Sciences, University of Florida, Gainesville, FL 32611, USA; 2 Department of Animal Sciences, State University of Maringa, Maringá, Brazil; 3 Department of Animal Breeding and Nutrition, Sao Paulo State University, Brazil

**Keywords:** amylase, butyrate, hemicellulase, *Saccharomyces cerevisiae*, xylanase

## Abstract

Bacterial cultures, enzymes, and yeast-derived feed additives are often included in commercial dairy rations due to their effects on ruminal fermentation. However, the effects of these additives when fed together are not well understood. The objective of this study was to evaluate the changes in ruminal fermentation when a dairy ration is supplemented with combinations of bacterial probiotics, enzymes and yeast. Our hypotheses were that ruminal fermentation would be altered, indicated through changes in volatile fatty acid profile and nutrient digestibility, with the inclusion of (1) an additive, (2) yeast, and (3) increasing additive doses. Treatments were randomly assigned to 8 fermenters in a replicated 4 × 4 Latin square with four 10 d experimental periods, consisting of 7 d for diet adaptation and 3 d for sample collection. Basal diets contained 52:48 forage:concentrate and fermenters were fed 106 g of dry matter per day divided equally between two feeding times. Treatments were: control (CTRL, without additives); bacterial culture/enzyme blend (EB, 1.7 mg/d); bacterial culture/enzyme blend with a blend of live yeast and yeast culture (EBY, 49.76 mg/d); and a double dose of the EBY treatment (2×, 99.53 mg/d). The bacterial culture/enzyme blend contained five strains of probiotics (*Lactobacillus animalis*, *Propionibacterium freudenreichii*, *Bacillus lichenformis*, *Bacillus subtilis*, and *Enterococcus faecium*) and three enzymes (amylase, hemicellulase, and xylanase). On d 8–10, samples were collected for pH, redox, volatile fatty acids, lactate, ammonia N, and digestibility measurements. Statistical analysis was performed using the GLIMMIX procedure of SAS. Repeated measures were used for pH, redox, VFA, NH_3_-N, and lactate kinetics data. Orthogonal contrasts were used to test the effect of (1) additives, ADD (CTRL vs. EB, EBY, and 2X); (2) yeast, YEAST (EB vs. EBY, and 2X); and (3) dose, DOSE (EBY vs. 2X). No effects (*P* > 0.05) were observed for pH, redox, NH_3_-N, acetate, isobutyrate, valerate, total VFA, acetate:propionate, nutrient digestibility or N utilization. Within the 24 h pool, the molar proportion of butyrate increased (*P* = 0.03) with the inclusion of additives when compared to the control while the molar proportion of propionate tended to decrease (*P* = 0.07). In conclusion, the inclusion of bacterial cultures, enzymes and yeast in the diet increased butyrate concentration; but did not result in major changes in ruminal fermentation.

## INTRODUCTION

The effects of bacterial cultures, enzymes, and yeast on dairy cattle have been evaluated and reviewed due to each one’s unique mode of action within the rumen and potential for increasing the health and productivity of animals (Beauchemin et al., 2003; Krehbiel et al., 2003; Desnoyers et al., 2009). The mode of action taken by bacterial cultures to alter ruminal fermentation is highly dependent on the species used. Lactate-producing and lactate-utilizing bacteria are often used due to their role in preventing acidosis, as the lactate-producing bacteria elevate the steady-state lactate concentrations to a level, which promotes increased growth of lactate-utilizing bacteria (Nocek et al., 2002). A few other mechanisms that have been outlined are the increase of propionate concentration through feeding *Propionibacterium* strains; and the inhibition of pathogen growth by competition for resources and attachment sites and an antibacterial effect from hydrogen peroxide production (Krehbiel et al., 2003).

Exogenous enzymes act on specific nutrients with xylanase and hemicellulase targeting the fiber fractions of the diet while amylase targets starch. The mode of action of enzymes can vary depending on where the activity occurs (Meale et al. 2014). The application of enzymes to the diet directly enhances enzymatic binding to substrates to form feed-enzyme complexes, which delay fermentation lag time within the rumen while also protecting them from proteolysis to improve the stability of the enzyme structure (Beauchemin et al., 2003). Once in the rumen, exogenous enzymes increase the rate of feed digestion and overall ruminal enzymatic activity and capacity (Meale et al., 2014).

The most commonly reported mode of action of *Saccharomyces cerevisiae* is the creation of a more anaerobic and stable environment, which promotes the growth of two key classes of ruminal bacteria: fibrolytic (Martin and Nisbet, 1992) and lactate-utilizing bacteria (Yoon and Stern, 1995). While studies do not always quantify the amount of fibrolytic enzymes, the rate of fiber digestion can increase with yeast supplementation (Dawson et al., 1990) indicating changes are occurring. The increase in lactate-utilizing bacteria leads to the stabilization of pH, prevention of lactate accumulation and an increase in VFA production (Chaucheyras-Durand et al., 2008). Live yeast may also be able to metabolize the lactate itself, further decreasing the concentration within the rumen and enhancing its effects (McAllister et al., 2011).

While the literature investigating each of these additives is rather extensive, the knowledge base is more limited on how combining them together affects ruminal fermentation. In a recent study by Oh et al. (2019), direct-fed microbials (yeast and bacteria) and enzymes were tested, and while both increased VFA production, they were not combined in a single diet to investigate the potential benefits of being fed together. Mixtures of bacteria cultures and yeast have been studied as direct-fed microbial (DFM) in a series of experiments which showed they prevent decreases in pH and complications from acidosis in high-risk diets (Nocek et al., 2002) as well as increase fiber digestion (Nocek and Kautz, 2006). When the DFM was fed to cows throughout the entirety of the transition period, cows consumed more feed and had greater milk production (Nocek et al., 2003). The different mechanisms used by these additives, and the current examples of their effects, may lead to an additive benefit from their combined effects. It is common practice for dairy nutritionists in the United States to use a combination of feed additives, including bacterial cultures, enzymes, and yeast due to the apparent effects they have on ruminal fermentation. However, to the best of our knowledge, the effects of combining these additives have not been scientifically tested.

Therefore, the objective of this study was to evaluate how ruminal fermentation patterns change when the diet was supplemented with combinations of yeast, bacteria and enzymes. Our hypotheses were that ruminal fermentation would be altered, indicated by changes in VFA profile and nutrient digestibility, with the: (1) inclusion of any additives; (2) the inclusion of yeast, and (3) an increased dosage of the three additives.

## MATERIALS AND METHODS

The animal handling and care procedures used in this study were conducted under protocols approved by the University of Florida Institutional Animal Care and Use Committee.

### Experimental Design and Diets

This experiment was a replicated 4 × 4 Latin square design with a completely randomized arrangement of treatments. Treatments were: (1) control without additives (CTRL); (2) bacterial culture and enzyme blend (EB); (3) bacterial culture and enzyme blend with a live yeast and yeast culture blend (EBY); and (4) double dose of bacterial culture and enzyme blend and the yeast products blend (2X). The same basal TMR (Table 1) was used for all treatments and was formulated to meet the NRC (2001) recommendations for a high producing lactating Holstein cow with 680 kg body weight and daily milk production of 45 kg. Prior to the experiment, the corn silage was dried for 72 h at 60 °C in a forced-air oven (Heratherm, Thermo Scientific, Waltham, MA) until it reached 90% DM. All dietary ingredients used to feed the fermenters were ground to pass through a 2 mm screen in a Wiley Mill (model #2; Arthur H. Thomas Co., Philadelphia, PA). The bacterial culture/enzyme blend contained five strains of probiotics with a combined 1× 10^9^ CFU (*Lactobacillus animalis, Propionibacterium freudenreichii, Bacillus lichenformis, Bacillus subtilis*, and *Enterococcus faecium*) and three enzymes (amylase [27,837 U/min/g amylose], hemicellulase [55.33 U/min/g xylose; 638.8 U/min/g mannose], and xylanase [58,598 U/min/g]). Enzyme activity is expressed in U, and one U defined as 1 μmol of substrate released per minute. The enzymes were derived from *Aspergillus oryzae* and *Trichoderma reeser*. The yeast component contained a mixture of live and culture yeast of the TS20 strain *Saccharomyces cerevisiae* with a CFU of 4.0 × 10^10^. Treatments were fed at the following doses: EB at 1.7 mg, EBY at 49.76 mg and 2X at 99.53 mg/d (Table 2). The doses were selected according to manufacturer guidelines and are comparable to the ones used in other studies (Oeztuerk et al., 2005; Leicester et al., 2016; Oh et al., 2019) as well as reflecting practical feeding protocols currently used by dairy nutritionists in the United States.

**Table 1.** Ingredient and chemical compositions of the experimental basal diet

Item (% DM)	Basal diet
Ingredient	
Corn silage	45.0
Grass hay	7.0
Ground corn grain	27.0
Soybean meal	20.5
Mineral mix	0.5
*Chemical composition*	
Crude protein	16.4
Neutral detergent fiber	28.3
Starch	30.5
Ether extract	2.2

**Table 2.** Experimental treatments

	Treatment^*a*^			
Additive, mg/d	CTRL	EB	EBY	2X
Bacteria^*b*^	.	0.74	0.74	1.484
Enzymes^*c*^	.	0.95	0.95	1.908
Yeast^*d*^	.	.	48.07	96.142
Total	.	1.70	49.77	99.534

^*a*^CTRL: control; EB: enzymes and bacteria; EBY: enzymes, bacteria and yeast; 2X: twice the dose rate of EBY treatment.

^*b*^Bacteria strains included in the pack were *Lactobacillus animalis*, *Propionibacterium freudenreichii*, *Bacillus lichenformis*, *Bacillus subtilis*, and *Enterococcus faecium*.

^*c*^Enzyme pack included 0.58 mg hemicellulose, 0.21 mg xylanase, and 0.16 mg amylase.

^*d*^Yeast contained 10.49 mg live yeast and 37.58 mg yeast culture both derived from *Saccharomyces cerevisiae*.

Each fermenter was fed 106 g/d DM distributed equally between two feedings one at 0800 h and the other at 1800 h. The yeast products and hemicellulase were added as dry products to their respective diets and divided into two equal doses. The bacteria culture and remaining enzymes were added to distilled water solutions to ensure accurate dosing due to the small amounts needed in the diet. Fresh solutions were prepared at 0700 h every day and were pipetted into the fermenters immediately before both morning and evening feedings.

### Dual-Flow Continuous Culture System

A dual-flow continuous culture system similar to that described by Hoover et al. (1976) and validated by Brandao et al. (2020) was used in this experiment. The conditions for all fermenters were constant with a temperature of 39 °C, continuous N_2_ gas infusion into both the headspace and fluid to maintain an anaerobic environment, and continuous agitation of 100 rpm. Artificial saliva (Weller and Pilgrim, 1974) was infused at a constant rate of 3.05 mL/min, allowing for passage rates to be individually controlled at an 11%/h liquid flow, and a 5.5%/h solid flow, to simulate a high producing dairy cow. These were collected in separate effluent containers.

### Experimental Period and Sample Collections

For this experiment, four 10-d periods, 7 d for adaptation and 3 d for sample collection, were used. Prior to inoculation with ruminal contents, fermenters were pre-warmed and under the continuous flush of N_2_ gas, and artificial saliva flow rates were established. On the first day of each period, fermenters were inoculated with ruminal contents collected from two cannulated mid-lactation Holstein cows consuming a diet similar to that which was fed to the fermenters. Ruminal contents were collected 2 h after the morning feeding and were strained through four layers of cheesecloth into pre-warmed thermos containers. The contents were immediately returned to the lab where a 50:50 mixture of ruminal contents from both cows was added to each of the fermenters. Each fermenter was inoculated with approximately 1.82 L of ruminal liquid, which allows it to clear the overflow spout.

Throughout the entirety of the experimental period, liquid and solid effluent weights were recorded daily prior to morning feeding. On d 5 of each period, liquid and solid effluent were pooled, mixed, and sampled to establish the background ^15^N abundance for each fermenter. During this time, a background sample was also collected for artificial saliva. A pulse dose of 0.1733 g (^15^NH_4_)_2_SO_4_ 10.2% atom excess (Sigma-Aldrich Co.) was infused into each fermenter to create a steady-state of ^15^N. Then (^15^NH_4_)_2_SO_4_ was continuously added to the system as a marker in the artificial saliva at a rate of 0.077 g/L until the end of each experimental period.

From d 8 to d 10, solid and liquid effluent containers were immersed in a water bath maintained at 1°C to prevent further microbial fermentation. On d 8, d 9, and d 10 effluent samples were collected for estimation of nutrient digestibility. Liquid and solid effluents were combined by fermenter and a subsample stored at –20 °C for further analyses of DM, CP, NDF, starch and ^15^N enrichment. Samples for VFA, NH_3_-N, and lactate analyses were also collected from the effluent containers, with 10 mL of VFA and NH_3_-N samples being acidified with 100 µL of 50% H_2_SO_4_ prior to all being frozen at –20 °C until further processing.

During the sampling period, pH and redox potential were measured using a portable pH meter (Thermo Scientific Orion Star A121) and recorded for each fermenter at 0, 1, 2, 4, 6, 8, and 10 h after morning feeding. On these same days, samples were collected at 0, 1, 2, 4, 6, and 8 h after feeding on inside the fermenter and strained through four layers of cheesecloth. During the sampling for VFA and NH_3_-N, 10 mL of mixed digesta was collected and immediately acidified with 100 µL of 50% H_2_SO_4_. The sample for lactate analysis was collected in a separate tube and all were frozen at –20 °C immediately.

On the final day of each period, all contents of the fermenter were centrifuged to isolate a bacterial pellet following a method modified from the one outlined by Krizsan et al. (2010). Briefly, fermenter contents were blended for 30 s, squeezed through four layers of cheesecloth and then washed with 400 mL of saline solution (0.9% NaCl). The filtered sample was then centrifuged (Sorvall RC-5B Refrigerated Superspeed Centrifuge, DuPont Instruments) three times. The first centrifugation was performed at 1,000 × *g* for 10 min and the residual feed particles were discarded. The residual supernatant was centrifuged at 11,250 × g for 20 min to obtain the bacterial pellet. The supernatant was discarded, and the bacterial pellet was resuspended in 200 mL of McDougall’s solution for final centrifugation at 16,250 × g for 20 min. The resulting bacterial pellet was transferred to a new container and stored at –20° C until further total N and N^15^ analysis, as well as DM and ash analysis.

All saliva, N^15^ background, digesta, and bacteria samples were freeze-dried. Once dried, first a mortar and pestle were used to grind the samples, and those being used for ^15^N analysis were then ball-milled at 25 Hz for 10 min using a Mixer Mill MM400 (Retsch, Newton, PA, USA).

### Chemical Analysis

Nutrient composition of feeds was determined from samples ground through a 1-mm screen. Diet ingredients and freeze-dried digesta samples were analyzed for DM (AOAC, 1990; method 930.15); ash (AOAC, 1990; method 942.05); NDF (Van Soest et al., 1991) with heat-stable α-amylase and sodium sulfite modified for Ankom^200^ Fiber Analyzer (Ankom Technology, Macedon, NY); starch (Hall 2015); and total N (AOAC, 2000; method 990.03) using rapid combustion with a micro elemental N analyzer (Vario Micro Cube, Elementar, Hanau, Germany).

Ruminal fluid samples collected for VFA and NH_3_-N analyses were thawed at room temperature, centrifuged at 10,000 × *g* for 15 min and the supernatant was collected for analysis. Samples were analyzed for NH_3_-N using the method described by Broderick and Kang (1980), using the phenol-hypochlorite method, and adapted to a 96-well flat-bottom plate. Analysis of total and individual VFA was determined via gas chromatography (Agilent 7820A GC, Agilent Technologies, Palo Alto, CA) with a flame ionization detector and a capillary column (CP-WAX 58 FFAP 25 m 0.53 mm, Varian CP7767, Varian Analytical Instruments, Walnut Creek, CA) that was maintained at 110°C, with injector temperature at 200 °C and detector at 220 °C.

Following the initial centrifugation, samples for VFA analysis were further processed following the method of Ruiz-Moreno et al. (2015) by adding a crotonic acid and metaphosphoric acid solution to the supernatant and freezing overnight. The sample was then centrifuged again at 10,000 × *g* for 15 min and the supernatant was mixed with ethyl acetate in a 2:1 ratio of ethyl acetate to the supernatant, vortexed and allowed to settle with the top layer being transferred to a chromatography vial for analysis.

Following ball-milling, samples of background, effluent and bacteria for ^15^N analysis were weighed into 8 × 5 mm pressed standard-weight tin capsules using a micro-balance (Excellence Plus XP Micro Balance, Mettler-Toledo GmbH, Laboratory & Weighing Technologies, CH-8606 Greifensee, Switzerland). Then 35 µl of a K_2_CO_3_ solution (10 g/L) was added to each sample and dried overnight at 40 °C to allow the complete evaporation of residual NH_3_-N. Samples were analyzed for DM (AOAC, 1990; method 930.15); ash (AOAC, 1990; method 942.05); and concentration of total N (AOAC, 2000; method 990.03) by rapid combustion with a micro elemental N analyzer (Vario Micro Cube, Elementar, Hanau, Germany). The % atom ^15^N was determined using isotope ratio mass spectrometry and reported as the fractional abundance of isotopes (^14^N/^15^N) × 100.

### Calculations

Flow of bacterial N and bacterial efficiency were determined as follows:

$$\begin{array}{l} \begin{array}{ll} & Bacterial\;N\;flow\;\left(expressed\;in\;g/d\right)\;\\ & =\;(NAN\;flow\times \%\;atom\;excess\;of^{15}N\;of\;NAN\;ef\;fluent)\;/\\ & \;(\%\;atom\;excess\;of^{15}N\;of\;bacteria\;pellet), \end{array} \end{array}$$

where % excess of ^15^N of NAN effluent is the result of subtracting % atom ^15^N in the background from the % atom excess of ^15^N of NAN effluent (Calsamiglia et al., 1996)

Flows of NH_3_-N and NAN as well as N metabolism were determined as follows (Bach and Stern, 1999):

$$\begin{array}{l} \begin{array}{ll} & NH_{3}-N\;flow\;\left(g/d\right)\;\\ & =\;NH_{3}-N\;concentration\;in\;effluent\;\left(mg/dL\right)\;\\ & \;\;\;\;\;\times \;\left(mL\;of\;total\;ef\;fluent\;flow/100\right), \end{array} \end{array}$$

$$\begin{array}{ll} NAN\;flow\;\left(g/d\right)\;= & \;g\;of\;total\;N\;in\;effluent\;\\ & -\;g\;of\;effluent\;NH_{3}-N \end{array}$$

$$\begin{array}{ll} & Nonmicrobial\;nonammonia\;N\;flow\;\left(g/d\right)\;\\ & \;=\;g\;of\;NAN\;in\;effluent\;\\ & \;\;-\;g\;of\;bacterial\;N\;in\;effluent \end{array}$$

$$\begin{array}{lll} Bacterial\;efficiency\;= & \;g\;of\;bacterial\;N\;flow\;/ & \\ & \;kg\;of\;OM\;truly\;digested \end{array}$$

$$\begin{array}{lll} & Efficiency\;of\;N\;use\;\left(ENU\right) & \\ & \;=\left(g\;of\;bacterial\;N/g\;of\;available\;N\right)\times 100 \end{array}$$

True digestibility of nutrients (OM, CP, NDF, and starch) was estimated using methods according to Soder et al. (2013):

$$\begin{array}{l} \begin{array}{l} True\;nutrient\;digestibility\;\left(\%\;DM\;basis\right)\;\\ =\;100\;\times [g\;of\;nutrient\;intake\;-\;(g\;of\;nutrient\;in\;effluent\\ \;-g\;of\;nutrient\;in\;saliva\;-\;g\;of\;nutrient\;in\;bacteria)]\\ \;\;\;÷\;g\;of\;nutrient\;intake \end{array} \end{array}$$

### Statistical Analysis

Statistical analysis was conducted using the GLIMMIX procedure in SAS 9.4 (SAS Institute Inc., Cary, NC). Data were analyzed as a 4 × 4 Latin square design. The model included treatment as a fixed effect and the random effects of square, period and fermenter within square included in the model. Additionally, for the pH, redox, VFA, NH_3_, and lactate time course data, time was included in the model as a fixed effect and data were analyzed as repeated measures. Orthogonal contrasts were used to test the effects of (1) ADD—the control compared to all treatments with additives (CTRL vs. EB, EBY and 2X); (2) YEAST—treatment without yeast compared to those with yeast (EB vs. EBY and 2X); and (3) DOSE—the single dose of enzymes, bacteria and yeast compared to the doubled dose (EBY vs. 2X). Significance was declared at *P* ≤ 0.05 and a tendency was considered when 0.05 < *P* ≤ 0.10.

## RESULTS AND DISCUSSION

While time course data were collected, there was no time by treatment effects observed for any of the parameters, thus all data were presented as either the overall mean (pH and redox potential) or the 24 h pool values (lactate, NH_3_-N and VFA).

### Ruminal pH and Redox Potential

The addition of bacteria, enzymes and yeast to the diet had no effect on pH (Table 3). While there are studies where rumen pH increased with the inclusion of bacteria (Nocek et al., 2002a), other studies support the lack of effect observed in this study when either DFM (Raeth-Knight et al., 2007) or fibrolytic enzymes (Chung et al., 2012) were fed. In both of these studies, the diets fed were not intended to cause an acidotic challenge and while the Chung paper did feed a higher NDF diet (33.9% vs. 28.3%) than in the present study, the Raeth-Knight fed a similar NDF content (28.8% DM) but a lower starch level (24.1% vs. 30.5%). Furthermore, meta-analyses on the effects of live yeast and DFM show conflicting results for pH, with one concluding that these additives, in this case yeast, can increase ruminal pH (Desnoyers et al. 2009), but also noting that the differences are potentially small enough to not consistently be detected in other experiments. Overall, the main effects of DFM seem to be more pronounced when an animal is undergoing a ruminal pH challenge, such as acidosis (Chiquette, 2009), indicating there may be no additional benefits to feeding them outside of these periods. Because the goal of the present study was not to cause a pH challenge, the control diet maintained non-acidotic conditions (pH 6.15, SEM = 0.05) within the fermenter. Therefore, the tested additives do not seem to play a role in altering ruminal pH under non-acidotic conditions.

**Table 3.** Effect of bacterial culture, enzyme, and yeast additives on ruminal fermentation parameters in a dual-flow continuous culture system

	Treatment^*a*^					*P-*value^*b*^		
Item	CTRL	EB	EBY	2X	SEM	ADD	YEAST	2X
pH	6.15	6.11	6.16	6.17	0.05	0.94	0.31	0.84
Redox, mV	37.28	37.83	34.33	34.33	6.95	0.56	0.27	0.88
Total VFA, mM	101.99	107.70	103.00	104.58	9.43	0.30	0.23	0.67
VFA, % of total VFA								
Acetate	53.91	53.45	52.92	54.54	1.42	0.72	0.74	0.11
Propionate	25.03	23.28	24.49	23.38	3.43	0.07	0.38	0.20
Butyrate	15.25	17.54	16.85	16.12	1.28	0.03	0.15	0.39
Isobutyrate	0.67	0.66	0.71	0.69	0.06	0.58	0.25	0.62
Valerate	2.25	2.27	2.28	2.28	0.10	0.75	0.88	0.99
Isovalerate	2.03	2.10	2.07	2.15	0.09	0.44	0.95	0.50
Caproate	0.86	0.69	0.68	0.85	0.11	0.31	0.52	0.21
Acetate:propionate	2.23	2.33	2.24	2.36	0.21	0.24	0.76	0.17
Lactate, mM	0.27	0.29	0.26	0.27	0.02	0.61	0.17	0.42

^*a*^CTRL: no additives; EB: addition of enzymes and bacteria; EBY: addition of enzymes, bacteria and yeast; 2X-EBY: addition of enzymes, bacteria and yeast at double the EBY dosage.

^*b*^Orthogonal contrasts: ADD—CTRL vs. EB, EBY and 2X; YEAST—EB vs. EBY and 2X; and DOSE—EBY vs. 2X.

There was also no effect of treatment on redox potential (Table 3). Redox potential is related to the yeast component of the additive and its ability to help maintain an anaerobic environment. The potential mode of action being that with less oxygen present (indicated by a smaller redox potential value), bacteria which are particularly sensitive to it (i.e., cellulolytic bacteria), would be able to better function within the improved rumen environment (Newbold et al., 1996; Marden et al., 2008). Due to the difficulty in obtaining measurements, data pertaining to redox potential is not commonly reported (Marden et al., 2005). However, it has been reported that there was no effect of either live yeast or yeast culture on redox potential (Oeztuerk et al., 2005), showing that there may be more to the mode of action relating to yeast than the stabilization of the rumen environment.

### Volatile Fatty Acids and Lactate

There was no effect of treatment observed on the total VFA concentration in the fermenters. A similar lack of increase in total ruminal VFA concentrations in dairy cattle has been observed in studies testing enzymes (Chung et al., 2012), bacteria (Raeth-Knight et al., 2007; Philippea et al., 2017) and yeast (Chung et al., 2011). Overall, results vary regarding the effects of these components on ruminal fermentation parameters due to variations in diet composition, DMI, animal category, dose tested, and specific treatment. The diets in these cases were relatively similar forage to concentrate ratio, ranging from 50% to 55% forage. Since there was no effect on total VFA concentrations in the present study, all individual VFA data will be reported as the molar proportion of the total VFA (Table 3). There was an increase in butyrate proportion (ADD, *P* = 0.03) and a tendency for propionate to decrease (ADD, *P* = 0.07) with additives. There were no yeast or dose effects observed for any VFA parameters.

The shifts in VFA composition favoring some VFA over others are highly varied among studies using these additives with increases in the proportion of acetate (Philippea et al., 2017) and propionate (Chung et al., 2011) having been observed. In the present study, there was a tendency for propionate molar proportion to decrease, which is likely related to the increase in butyrate proportions. While it is common to see an increase in propionate when feeding certain bacteria, such as the *Propionibacterium* fed in this study, this was not the case in the present study.

While it is less common to observe an increase in ruminal butyrate compared to propionate and acetate, increasing butyrate has been reported with the addition of probiotics (Chiquette, 2009) and with a combination of enzymes (Zilio et al., 2019). The increase in butyrate may be tied to the presence of lactate-utilizing bacteria, which produce butyrate as an end product during the conversion from lactate to pyruvate (Goad et al., 1998), and was fed as part of the bacterial component of this experiment. Amylolytic enzymes, such as those fed in this study, may also increase butyrate due to a shift in the type of bacteria growing to those that produce butyrate as their predominant end product. While these bacteria themselves are not amylolytic, they may be utilizing cross-feeding, that is using the products from those that are able to ferment the main substrate of starch (Tricarico et al., 2008). In a study comparing live and autoclaved yeast products, both increased butyrate production; however, the autoclaved yeast had a greater effect potentially due to the components being used as a substrate for microorganisms, which produce butyrate (Oeztuerk et al., 2005).

There were no effects on lactate concentrations (Table 3). The inclusion of lactate-producing bacteria allows the rumen environment to potentially adjust to the constant levels of lactate thus increasing by promoting an increase in the amount of native lactate-utilizing bacteria present to utilize lactate when there is a dietary-linked increase (Ghorbani et al., 2002). In regards to yeast, there are two potential modes of action for the reduction of lactate concentrations within the ruminal contents. The first being that it promotes the use of lactate by other bacteria through its effects on the environment. The second being that the yeast itself uses lactate (Desnoyers et al., 2009). When yeast and bacteria were combined, the risk for lactate-related acidosis was reduced (Nocek et al., 2002). However, other studies have not observed any changes in ruminal lactate concentrations (Chung et al., 2011, Philippea et al., 2017). In a study testing the effects of two different strains of *Saccharomyces cerevisiae* during a subacute ruminal acidosis challenge, differences were observed in how they altered ruminal fermentation, with cows supplemented with strain 2 experiencing a more acidic rumen environment (Chung et al., 2011). This indicates that not all yeast products are similar in their mode of action, even when they are from the same species, and may explain some of the variability across studies.

### Nutrient Digestibility

No effects of treatment were observed on the digestibility parameters measured of DM, OM, CP, NDF, and starch (Table 4). In the case of the current study, the control diet’s digestibility values fall within a reasonable range (DM = 61.9%; NDF = 55.8%), thus the diet quality may have allowed for adequate fermentation even without the inclusion of an additive. In a recent study comparing yeast and enzymes, but not in combination, neither influenced the apparent digestibility of any nutrients (Oh et al., 2019). When fibrolytic and amylolytic enzymes were fed in combination, no effect was found on apparent total-tract digestibility on any nutrient parameters (Zilio et al., 2019). However, nutrient digestibility does not seem to be the driving force behind changes in ruminal fermentation patterns since both of these studies did see changes within the VFA data, with an increase in total VFA (Oh et al., 2019) and an increase in butyrate (Zilio et al., 2019). All of this together could indicate that changes are occurring within the species present in the microbial community to drive this shift. Furthermore, this lack of effect of additives on nutrient digestibility is common throughout the literature and may be attributed to the vast differences in the products being tested, the doses at which they are applied and experimental conditions, as discussed in reviews (Beauchemin et al., 2003; Krehbiel et al., 2003; Desnoyers et al., 2009). It is possible that when extreme diets (i.e., high starch, high forage, or low-quality fiber) are used, that additives such as the ones tested in this study may play a greater role in ruminal nutrient utilization; however, the goal of this study was to evaluate these additives under commonly fed, high-quality diets, and based on our results these additives have a minor role in ruminal fermentation and ruminal nutrient utilization.

**Table 4.** Effect of bacterial culture, enzyme and yeast additives on true nutrient digestibility in a dual-flow continuous culture system

Digestibility, %	Treatment^*a*^					*P-*value^*b*^		
	CTRL	EB	EBY	2X	SEM	ADD	YEAST	DOSE
Dry matter	61.9	59.5	61.3	59.7	2.80	0.38	0.64	0.48
Organic matter	62.8	60.7	61.4	60.8	2.45	0.27	0.79	0.74
Crude protein	67.0	68.6	65.3	67.4	2.83	0.98	0.43	0.53
Neutral detergent fiber	55.8	52.0	53.7	52.4	2.98	0.15	0.64	0.59
Starch	94.8	93.8	92.8	94.2	0.88	0.14	0.74	0.15

^*a*^CTRL: no additives; EB: addition of enzymes and bacteria; EBY: addition of enzymes, bacteria and yeast; 2X-EBY: addition of enzymes, bacteria, and yeast at double the EBY dosage.

^*b*^Orthogonal contrasts: ADD—CTRL vs. EB, EBY, and 2X; YEAST—EB vs. EBY and 2X; and DOSE—EBY vs. 2X.

### N Flows and Metabolism

All data pertaining to N are presented in Table 5. No effects were observed on NH_3_-N flows. This finding matches that of Chung et al. (2012), who also did not observe any changes in NH3-N when feeding enzymes. Furthermore, no effect of enzymes (Oh et al., 2019; Zilio et al., 2019), yeast (Chung et al., 2011; Jiang et al., 2017), and bacteria (Raeth-Knight et al., 2007; Philippea et al., 2017) on NH3-N is consistently observed. In the present study, there were also no effects on any of the parameters used to observe N flow or metabolism. In their study, Zilio et al. (2019) also did not observe any changes in N balance or microbial CP values. The lack of difference in N utilization efficiency in that study was thought to be due to the proteolytic activity within the rumen. The degradation of exogenous enzymes by native proteolytic enzymes is a concern that has been raised before regarding their efficacy as a feed additive (Beauchemin et al., 2003). The observations from this study also suggest that the expected yeast-driven increase in microbial growth (Yoon and Stern, 1995) may not be occurring, but rather a change in the microbial community may be a more plausible explanation due to the change in the VFA profile.

**Table 5.** Effect of bacterial culture, enzyme and yeast additives on ruminal N flows and metabolism in a dual-flow continuous culture system

	Treatment^*a*^					*P-*value^*b*^		
Item	CTRL	EB	EBY	2X	SEM	ADD	YEAST	DOSE
NH_3_-N^*c*^, mg/dL	19.79	19.45	19.82	20.66	1.46	0.87	0.50	0.54
N flows, g/d								
Total N	2.59	2.43	2.74	2.5	0.18	0.77	0.13	0.11
NH_3_-N	0.76	0.71	0.78	0.77	0.08	0.79	0.13	0.53
NAN^*d*^	1.83	1.71	1.95	1.74	0.19	0.82	0.25	0.13
Bacterial N	0.91	0.84	0.99	0.84	0.14	0.80	0.45	0.17
Dietary N	0.92	0.87	0.97	0.91	0.08	0.98	0.43	0.53
RDP-N^*e*^ supply	2.49	2.52	2.43	2.49	2.32	0.92	0.46	0.55
RUP-N^*f*^	0.92	0.87	0.97	0.91	0.08	0.98	0.43	0.53
ENU^*g*^	32.72	30.20	35.47	30.04	5.07	0.80	0.45	0.17
Bacterial efficiency^*h*^	14.36	13.77	16.06	13.42	1.86	0.97	0.48	0.11

^*a*^CTRL: no additives; EB: addition of enzymes and bacteria; EBY: addition of enzymes, bacteria and yeast; 2X-EBY: addition of enzymes, bacteria, and yeast at double the EBY dosage.

^*b*^Orthogonal contrasts: ADD—CTRL vs. EB, EBY, and 2X; YEAST—EB vs. EBY and 2X; and DOSE—EBY vs. 2X.

^*c*^Ammonia nitrogen.

^*d*^Nonammonia nitrogen.

^*e*^Rumen degraded protein N.

^*f*^Rumen undegraded protein N.

^*g*^Efficiency of N use = g of bacterial N/g of available N.

^*h*^Bacterial efficiency = g of bacterial N/kg of OM truly digested.

## CONCLUSION

This study targeted evaluating the combination of these feed additives in high-quality dairy diets that are commonly fed across the US. In summary, there were no benefits to including these additives in the diet on ruminal fermentation, with the exception of an increase in the molar proportion of butyrate. Regarding the effects on ruminal fermentation, only an increase in the molar proportion of butyrate was observed. These findings will help direct future research aiming to better understand and narrow down the potential modes of action used by these feed additives. This experiment was able to isolate the rumen effects, removing potential confounding effects of differential DMI, rumen pool size, and nutrient concentration, indicating that the positive results seen in *in vivo* studies may be due to a mode of action that is separate from the rumen itself, such as DMI, intestinal absorption, or immune response related.
